# The nationality benefit: Long-term memory associations enhance visual working memory for color-shape conjunctions

**DOI:** 10.3758/s13423-021-01957-2

**Published:** 2021-06-22

**Authors:** Markus Conci, Philipp Kreyenmeier, Lisa Kröll, Connor Spiech, Hermann J. Müller

**Affiliations:** 1 Allgemeine und Experimentelle Psychologie, Department of Psychology, Ludwig-Maximilians-Universität München, München, Germany; 2 Graduate School of Systemic Neurosciences, Ludwig-Maximilians-Universität München, München, Germany; 3 Department of Ophthalmology and Visual Sciences, University of British Columbia, Vancouver, Canada; 4 Department of Psychology, Humboldt-Universität zu Berlin, Berlin, Germany; 5 Department of Psychology, University of Oslo, Oslo, Norway

**Keywords:** Visual working memory, Working memory capacity, Change detection, Visual search, Long-term memory, Familiarity, Object knowledge

## Abstract

Visual working memory (VWM) is typically found to be severely limited in capacity, but this limitation may be ameliorated by providing familiar objects that are associated with knowledge stored in long-term memory. However, comparing meaningful and meaningless stimuli usually entails a confound, because different types of objects also tend to vary in terms of their inherent perceptual complexity. The current study therefore aimed to dissociate stimulus complexity from object meaning in VWM. To this end, identical stimuli – namely, simple color-shape conjunctions – were presented, which either resembled meaningful configurations (“real” European flags), or which were rearranged to form perceptually identical but meaningless (“fake”) flags. The results revealed complexity estimates for “real” and “fake” flags to be higher than for unicolor baseline stimuli. However, VWM capacity for real flags was comparable to the unicolor baseline stimuli (and substantially higher than for fake flags). This shows that relatively complex, yet meaningful “real” flags reveal a VWM capacity that is comparable to rather simple, unicolored memory items. Moreover, this “nationality” benefit was related to individual flag recognition performance, thus showing that VWM depends on object knowledge.

## Introduction

Maintaining a stable representation of the visual environment in the absence of direct retinal stimulation requires that goal-relevant items are stored in a temporary buffer, referred to as visual working memory (VWM). Studies that investigate VWM often use the “change detection” paradigm (Luck & Vogel, 1997; Phillips, 1974), where observers are asked to remember a set of objects (usually colored squares) in an initial memory display. After a short retention interval, a probe item is shown and participants are required to indicate whether the probed item has changed relative to the object presented previously at the same location in the memory display. The typical finding is that about three to four objects can be maintained concurrently in VWM (Cowan, 2001; Luck & Vogel, 1997). However, this maximum capacity may vary quite substantially for different types of object. For instance, while the capacity for colored squares is some three to four items, it is only one or two for more complex objects (e.g., Chinese characters, polygons, shaded cubes; Alvarez & Cavanagh, 2004; Eng et al., 2005; Luria et al., 2010). The capacity reduction for complex objects may partly be owing to variations in inter-item-similarity, with more similar to-be-remembered items leading to elevated memory comparison errors (Awh et al., 2007). However, relatively regular (i.e., symmetric) objects nevertheless reveal a larger memory capacity than irregular objects even when controlling for similarity differences in the perceptual input (Chen et al., 2016; Chen et al., 2018). Together, this indicates that VWM is limited in capacity, with the overall number of items that can be retained varying for different types of (more or less complex) objects.

Seemingly at odds with this rather limited capacity of VWM is the subjective impression in everyday life that orienting in natural environments is not per se constrained by some upper limit. In fact, keeping track of objects in natural scenes has been shown to be far better than the capacity limits as estimated in change detection studies (Melcher, 2001). A potential enhancement of VWM capacity may, for instance, be rendered by invariant and structured object arrangements, which are typically also present in real-world environments (Chen et al., 2021; Conci & Müller, 2014; Hollingworth & Henderson, 2000; Nie et al., 2017; but see Quinlan & Cohen, 2016). Moreover, everyday objects usually also convey meaning, that is, they are linked to long-term memory (LTM) structures representing knowledge about these objects, such as in terms of categorical or semantic information. Thus, real-world objects retained in VWM might automatically invoke a representation of the object’s meaning in LTM, and such associations with stored knowledge might in turn improve VWM capacity. Previous studies have shown that VWM is indeed better for upright versus inverted faces (Asp et al., 2021; Curby & Gauthier, 2007; Scolari et al., 2008) and when the positioning of everyday objects accords with frequently experienced real-world regularities (Kaiser et al., 2015). Moreover, VWM consolidation of complex polygon shapes may be improved by familiarizing observers with these items (Blalock, 2015). More generally, expertise – such as for cars (Curby et al., 2009), pokémon characters (Xie & Zhang, 2017), or familiar faces of celebrities (Jackson & Raymond, 2008) – can enhance VWM capacity. Together, these findings indicate that meaning and familiarity may indeed improve VWM, suggesting that LTM interacts with the short-term retention of visual information.

The above examples illustrate that expertise for highly trained stimuli from specific object categories may enhance VWM performance. Brady et al. (2016) recently showed that a comparable improvement can also be observed for a variety of arbitrary, everyday real-world objects. In their study, a change detection paradigm was employed that presented meaningless colored squares (as a baseline) or, alternatively, photographs of meaningful objects (e.g., a cookie, a cup, a chair) as to-be-memorized items during a short (200 ms) or much longer (up to 2,000 ms) encoding phase. For the short encoding durations, the results revealed VWM capacity to be about three items, irrespective of the to-be-memorized stimulus. Interestingly, however, for the longer encoding durations, the real-world stimuli exhibited an increase in capacity; while there was also a (numerical) gain for the baseline condition (colored squares), the increase was significantly smaller (but see Quirk et al., 2020). Brady et al. (2016) took this pattern to indicate that LTM associations for everyday objects can improve VWM capacity, provided that observers have sufficient time to encode the presented objects in detail.

However, while meaning-related associations derived from LTM may indeed improve object representations in VWM, the different object types presented in Brady et al. (2016) differed not only in terms of their meaning, but also in their constituent perceptual features. Accordingly, follow-up studies attempted to match the perceptual input of meaningless and meaningful to-be-remembered stimuli by presenting scrambled (Asp et al., 2021; Brady & Störmer, 2021; Sahar et al., 2020) or warped (Stojanoski et al., 2019; Veldsman et al., 2017) versions of the real-world objects. The results consistently showed that meaningful objects were remembered better than perceptually matched, distorted items. However, these distortions, applied to a given object, would usually not only eliminate the object’s meaning, but also change the perceptual structure of the object itself, thereby potentially increasing the perceptual complexity of that item while also increasing its similarity to other to-be-remembered items. Enhanced VWM performance for real-world objects may therefore not be specifically related to object meaning; differences could also result (at least in part) from variations in the structure of an object and its resulting complexity (Alvarez & Cavanagh, 2004; Chen et al., 2018), and/or the degree of variability of the presented stimulus set (Awh et al., 2007; Brady & Störmer, 2020b).

**Fig. 1:**
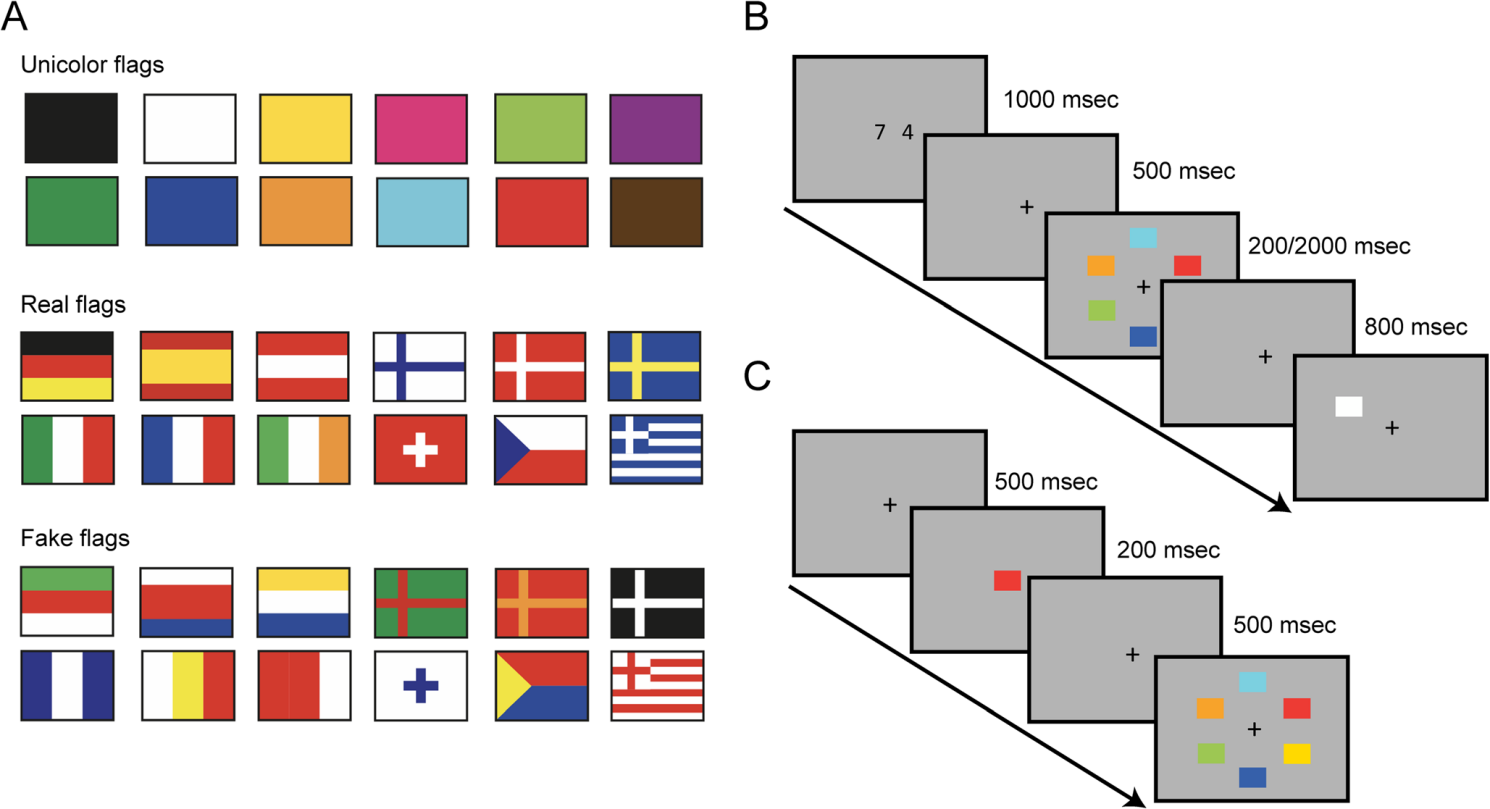
(**A**) The stimulus set used in this experiment. The unicolor flag stimuli (top) were used as a baseline measure to estimate visual working memory capacity. The real flag stimuli (middle) consisted of more complex color-shape conjunctions that represent official flags from the following European countries: Germany, Spain, Austria, Finland, Denmark, Sweden, Italy, France, Ireland, Switzerland, Czech Republic, and Greece (from top left to bottom right, respectively). The fake flags (bottom) presented identical colors and shapes but these were combined such that they did not resemble any actual, known flags. (**B**) Example trial sequence in the change detection task: A given trial started with the presentation of two to-be-remembered digits. After a brief delay, the actual memory display was presented. Subsequent to a short retention interval, a probe item was shown, which required a same/different response (in the example, the correct response would be “different”). (**C**) Example trial sequence in the visual search task, which presented an initial fixation cross, followed by a target probe. After a short delay, the search display was presented, to which observers gave a speeded response indicating whether the target was present (as in the example), or absent

The ultimate aim of the current study was to test whether meaning can influence VWM performance while maintaining the overall stimulus structure and thereby controlling the perceptual complexity of the to-be-remembered items. To ensure the latter, we compared relatively simple stimuli that invoke specific LTM associations to a perceptually identical and equally complex stimulus set devoid of any meaning. For this comparison, we designed a set of stimuli that resembled well-known, “real” European flags, and a set of visually similar, but entirely fictional, “fake” flags (the latter were generated from the very same set of colors and shapes, which were, however, rearranged to render meaningless configurations of a comparable structure).^1^ In addition, a third baseline condition presented colored rectangles (“unicolor” flags) to reflect the typical stimuli used in standard change detection tasks (see Fig. 1A). Thus, if meaningful objects indeed improve VWM independently of perceptual complexity, the gains would be expected to be greater for real than for fake flags.

## Methods

The experiment comprised three parts. Part 1 consisted of a change detection task (e.g., Luck & Vogel, 1997): observers were required to memorize a set of stimuli and report (after a short retention interval) whether a subsequently presented probe stimulus was the same as or different from the item presented initially at the same location (see Fig. 1B).

In Part 2, observers performed a visual search task, in order to quantify the information load per item for a given type of flag stimulus (cf. Alvarez & Cavanagh, 2004). To ensure that the estimation of the information load was not biased by the previous exposure to the very same stimuli in the change detection task, the search task was tested in two separate samples of observers: those who had previously participated in the change detection task (main experiment), and a second group who only performed the search task (control experiment). As illustrated in Fig. 1C, observers were first presented with a to-be-searched-for target stimulus; this was followed by a search display composed of either three or six items (the display size) from the sets of real, fake, or, respectively, unicolor flags, to which observers were required to issue a speeded target absent/present response. The information load for each type of stimulus was quantified by computing the mean search rates (i.e., the slopes of the functions relating display size to the response times (RTs)).

The final part, Part 3 was performed to ascertain that the real and fake flag stimuli were or, respectively, were not linked to a specific memory association. To this end, observers were administered a questionnaire (at the end of the experiment) designed to quantify their knowledge about both sets of flags.

### Participants

Twenty-five adults (eight male, mean age: 25.6 years) participated in the experiment for payment of 9 €/h. All participants had normal or corrected-to-normal vision and all but two were right-handed. A second sample of 25 adults (11 male, mean age: 29.5 years, 24 right-handed) participated in the additional control experiment, which only presented the visual search task. All observers provided written informed consent, and the experimental procedure was in accordance with the Declaration of Helsinki and approved by the ethics committee of the Department of Psychology, LMU Munich.

The sample size was determined based on the effect sizes derived from previous, comparable studies, in particular that of Brady et al. (2016), who tested 12–18 participants per experiment. On the basis of this study, a power analysis revealed that in order to detect an f(U) effect size of 0.35 with a power of 80% and an alpha of .05, a sample of 15 participants would be required. We further increased (i.e., almost doubled) our sample to N=25 observers to ensure sufficient statistical power to detect a difference across the three types of flag.

### Apparatus and stimuli

Stimulus presentation and data collection was controlled by a Windows PC running Matlab and Psychophysics toolbox extensions (Brainard, 1997; Pelli, 1997). All stimuli were presented on a grey background (8.31 cd/m^2^) on a 20-in. CRT monitor (1,920 × 1,080 pixels screen resolution, 80-Hz refresh rate) at a viewing distance of approximately 60 cm. Each stimulus subtended 1.4° × 1.0° of visual angle. A black fixation cross (0.6°) was presented at the center of the screen and stimuli were evenly arranged along an imaginary circle (radius = 5.7°) around fixation. Three different sets of stimuli, or “flag types,” were presented, each consisting of 12 different color-shape configurations (see Fig. 1A). First, “unicolored flags” consisted of flag-shaped rectangles that presented a single, highly discriminable color (black, white, pink, yellow, light and dark green, purple, light and dark blue, orange, red, and brown). In addition, two sets of more complex color-shape configurations were presented, which in the case of “real flags” resembled the official flags of 12 well-known European countries (Germany, Spain, Austria, Finland, Denmark, Sweden, Italy, France, Ireland, Switzerland, Czech Republic, and Greece). In addition, the set of “fake flags” were composed of the very same colors and shapes as used for the real flags, but these were arranged in different color and shape combinations, yielding 12 largely meaningless configurations of similar complexity. Thus, real and fake flags were composed of identical shapes and colors, but differed solely in terms of their meaningful association with a European country. The unicolor flags in turn were used as a baseline measure to assess working memory capacity with the stimulus material typically used in change detection tasks (e.g., Luck & Vogel, 1997).

### Procedure

In the experiment, observers first completed the change detection task, followed by the visual search task. Each task started with a short practice block of 16 trials, followed by 384 experimental trials (see details below). After the experiment, each participant completed a questionnaire that presented the real and fake flag stimuli. Observers had to indicate whether they associated a given flag stimulus with a known country (yes/no recognition). If they responded “yes,” they were asked to additionally provide the associated country’s name (recall). The entire experiment took approximately 1.5 h to complete.

An example trial sequence in the *change detection task* is depicted in Fig. 1B. To prevent the use of verbalization strategies, participants were asked to silently rehearse two digits that were presented at the beginning of each trial for 100 ms. Next, a fixation cross was presented for 500 ms, after which a memory display was shown. A given display consisted of six different stimuli from one set (either unicolor, real, or fake flags), which were randomly selected from a given set and presented in circular arrangement around fixation for an exposure time of either 200 or 2,000 ms. After a retention interval of 800 ms, a probe stimulus appeared. On “no-change” trials, this probe would present the same item that had previously been shown at the exact-same location in the memory display; on “change” trials, by contrast, the probe depicted a new, randomly selected item from the same stimulus set as in the memory display (but which had not been presented in the previous memory display). The probe remained visible until participants responded. Participants responded with the left and, respectively, the right mouse key to indicate whether the probe object was the same as or different from the object at the same location in the previous memory display (see Fig. 1A; in the example, the correct response would be “different”). Observers were asked to respond as accurately as possible; there was no stress on response speed. Finally, after the response to the probe, observers were asked to enter the memorized digits on the keyboard (shown at the beginning of a trial). Trials were separated from each other by an interval of 1,000 ms.

The same stimuli were also presented in a subsequent *visual search task,* in order to estimate the search efficiency for the three flag types (see Fig. 1C for an example trial sequence). Note that this search task was also tested in an additional, independent sample of observers who did not perform the other parts of the experiment. Each trial presented an initial fixation cross for 500 ms, followed by the presentation of the target stimulus at the center of the screen for 200 ms. After a 500-ms delay, the actual search display was shown, which consisted of either three or six different stimuli, chosen randomly from one stimulus set, and arranged around the central fixation cross. In case of the three-item display, stimuli were presented in an upright or downward pointing triangle arrangement (with equal probability). Participants had to respond as quickly and as accurately as possible, indicating whether the prespecified target was absent or present, with left and right mouse keys, respectively (in the example display in Fig. 1C, the correct response would be “target-present”). In case of an erroneous response, feedback was provided by presenting the word “error” for 1,000 ms in the center of the screen. Search displays remained visible until a response was issued. The inter-trial interval was 1,000 ms.

### Design

In the change detection task, three experimental factors were systematically varied: flag type (unicolor, real, fake flags), change (yes, no), and presentation duration (200, 2,000 ms). The visual search task also varied three factors: flag type (unicolor, real, fake flags), target (absent, present), and display size (three, six items). In both tasks, different types of flags were presented in separate blocks, with block order randomized (see Chen et al., 2016, for a similar procedure). All combinations of the factors change and presentation time (in the change detection task) or target and display size (in the search task) were presented with equal probability and in random order within each block. Each task consisted of 12 blocks of 32 trials each, resulting in 384 experimental trials in total per task.

## Results

### Change detection

**Fig. 2:**
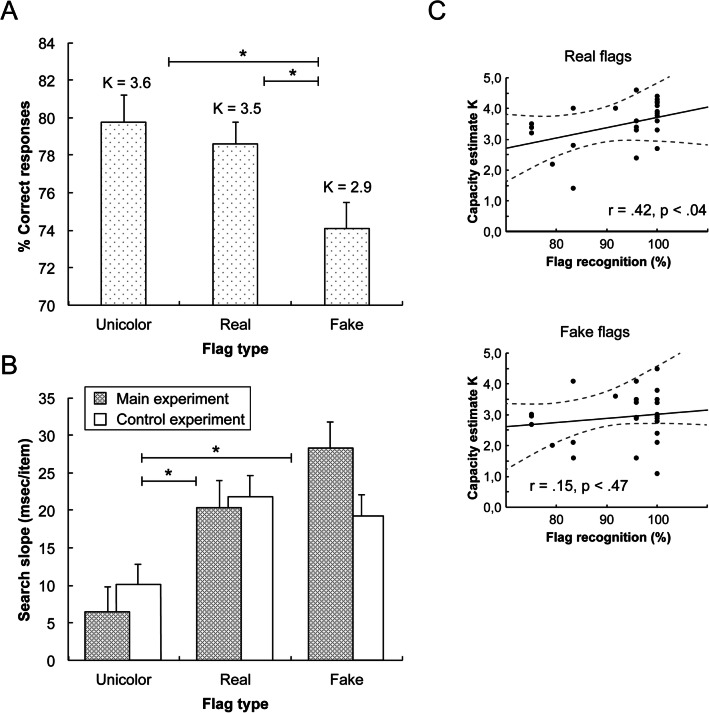
(**A**) Mean percentage of correct responses as a function of flag type in the change detection task. The numbers above each bar present the corresponding estimates of visual working memory capacity K. (**B**) Mean search slopes (in msec per item) for each of the three flag type conditions in the visual search task in two groups of observers, as tested in the main experiment (gray bars) and in the control experiment (white bars). Asterisks depict significant differences between pairwise comparisons. Error bars represent the standard error of the mean. (**C**) Correlation between individual flag recognition performance (% correct) and working memory capacity estimates K. The graphs depict the significant relationship for real flags (top), and the non-significant relationship for fake flag stimuli (bottom). The dashed gray lines denote the 95% confidence interval

Figure 2A presents the mean percentage of correct responses for the different types of flag. In addition, Table 1 also provides the corresponding mean values for each flag type condition across the two presentation times of the memory display. A repeated-measures analysis of variance (ANOVA) on the accuracy data was performed with the factors flag type (unicolor, real, fake flags) and presentation time (200 vs. 2,000 ms). We additionally report the corresponding Bayes factors (BF_10_) as revealed by comparable Bayesian statistics using JASP (JASP Team, 2017). The Bayes factor provides the ratio with which the alternative hypothesis is favored over the null hypothesis: larger BF_10_ values argue in favor of the alternative hypothesis, with values above 3 denoting “substantial evidence” in favor of the alternative hypothesis; values less than 1 favor the null hypothesis; see Dienes (2011). This analysis yielded significant main effects of flag type, F(2, 48) = 13.12, p < .001, η_p_^2^ = .35, BF_10_ = 81.17, and presentation time, F(1, 24) = 70.13, p < .001, η_p_^2^ = .75, BF_10_ > 100. The unicolor (baseline) flags (79.8%) and the real flag stimuli (78.6%) yielded significantly higher accuracies than the fake flags (74.1%), ts > 3.91, ps < .002, ds > .78, BF_10_s > 51.06, but unicolor and real flags did not differ from each other, t(24) = 1.13, p = .27, d_z_ = .23, BF_10_ = 0.37. In addition, increasing the duration of the memory display from 200 to 2,000 ms resulted overall in more accurate responses (73.4% vs. 81.6%). The interaction effect was not significant, F(2, 48) = 0.59, p = .55, η_p_^2^ = .02, BF_10_ = 0.18.

**Table 1:** Mean accuracies (percent correct) and corresponding estimates of working memory capacity K for the three types of flag across presentation times in the change detection task

	Flag type	Presentation time (ms)	
		200	2,000
% correct	Unicolor	75 [1.7]	85 [1.6]
	Real	75 [1.4]	82 [1.5]
	Fake	70 [1.4]	78 [1.7]
K estimates	Unicolor	3.0 [0.21]	4.1 [0.19]
	Real	3.0 [0.16]	3.9 [0.18]
	Fake	2.4 [0.16]	3.4 [0.21]

Values in square brackets depict the standard error of the mean

We also estimated the number of individual objects remembered using Cowan’s K (Cowan, 2001): K = (H – FA) × N, where K is the number of items stored, H is the hit rate, FA the false alarm rate, and N the number of items presented. A comparable ANOVA to the previous one on the K estimates again revealed significant main effects of flag type, F(2, 48) = 12.84, p < .001, η_p_^2^ = .35, BF_10_ = 72.17, and presentation time, F(1, 24) = 70.17, p < .001, η_p_^2^ = .75, BF_10_ > 100, but again no reliable interaction, F(2, 48) = 0.62, p = .54, η_p_^2^ = .03, BF_10_ = 0.17. K values were comparable for unicolor (3.6) and real flags (3.5), t(24) = 0.98, p = .33, d_z_ = .19, BF_10_ = 0.32, but both yielded larger capacity estimates than fake flags (2.9), ts > 3.94, ps < .001, ds > .78, BF_10_s > 53.70. Moreover, longer presentation times again led to an overall increase of the K estimate (2.8 vs. 3.8 for the 200- vs. 2,000-ms presentation times).^2^

Finally, observers performed the digit memory task (used to prevent verbal rehearsal) with near-ceiling accuracy (94.5% correct responses). The individual digit report accuracy was not significantly correlated with the K estimates in either of the three flag conditions, rs < .19, ps > .35, all BF_10_ < 0.38.

### Visual search

The visual search task was performed in order to obtain an independent measure of search efficiency as an estimate of the visual information load posed by each type of stimulus (see Alvarez & Cavanagh, 2004). To ensure that this estimate was unaffected by participants having performed the main, VWM experiment, we recruited a second, control group of observers who only performed the search task. Search efficiency was calculated by computing the slope of the function relating the detection latencies, that is, the mean RTs (in ms), to display size. Steeper slopes would be indicative of reduced search efficiency, providing evidence for an increased information load. Outliers (RTs below 300 and above 3,000 ms) and error responses were excluded from the data proper prior to the slope analysis (overall 5.5% of all trials). Figure 2B presents the mean search slopes (in ms/item) for each of the three types of flag in the main and control experiments (gray and white bars, respectively). For the main experiment, a repeated-measures ANOVA on the slopes with the single factor Flag Type (unicolor, real, fake flags) revealed a significant effect, F(2, 48) = 12.29, p < .001, η_p_^2^ = .34, BF_10_ > 100. Search was more efficient in the unicolor flag condition compared to both the real and the fake flag conditions, ts > 2.99, ps < .007, ds > .60, BF_10_s > 7.25 (mean slopes were 7, 20, and 28 ms/item, respectively). Furthermore, search appeared slightly more efficient in the real as compared to the fake flag condition, evidenced by a marginal (though in terms of the BF inconclusive) effect, t(24) = 2.01, p = .056, d_z_ = .40, BF_10_ = 1.17. The (search-task-only) control group showed a similar pattern: a significant flag-type effect, F(2, 48) = 4.88, p < .02, η_p_^2^ = .17, BF_10_ = 5.85, due to more efficient search with unicolor flags than with both real and fake flags, ts > 2.23, ps < .04, ds > .44, BF_10_s > 1.78 (mean slopes were 10, 21, and 19 ms/item, respectively), without a slope difference between the latter two flag types, t(24) = 0.67, p = .51, d_z_ = .13, BF_10_ = 0.25. Overall, this indicates that the information load across both experiments was similarly high for real and fake flags, t(49) = 0.99, p = .32, d_z_ = .14, BF_10_ = 0.24, but higher for these than for unicolor flags, ts > 4.29, ps < .001, ds > .60, BF_10_s > 270.

The mean error rate in the visual search task was 4.5% in the main experiment and 5.8% in the control experiment. A repeated-measures ANOVA on the error rates with the factor flag type revealed no significant difference between conditions in either experiment, F(2, 48) > 0.57, p < .55, η_p_^2^ > .03, BF_10_ > 0.18, that is, there was no evidence of a trade-off between the pattern of errors and RT slope measures.

### Flag memory

The questionnaire, which was intended to assess participants’ knowledge about the presented flag stimuli, revealed that observers were highly accurate in discriminating the real from the fake flags (mean recognition accuracy: 92.8%). Moreover, they were very accurate in recalling the correct country for a given real flag stimulus (mean recall accuracy: 79.0%), indicating that the real flag stimuli could be reliably associated with meaning. Conversely, erroneous recall of a country for a fake flag only occurred in 1.0% of the respective questionnaire items. An additional correlational analysis showed that the individual K estimates for the real flag condition were significantly correlated with both mean flag recognition accuracy, r = .42, p < .04 BF_10_ = 3.88 (see Fig. 2C, top panel) and mean recall accuracy for the real flags, r = .45, p < .03, BF_10_ = 5.36. In contrast, individual K estimates for the fake flag condition exhibited no significant correlation with mean flag recognition accuracy, r = .15, p = .47, BF_10_ = 0.47 (see Fig. 2C, bottom panel) or with mean recall accuracy for fake flags, r = -.12, p = .54, BF_10_ = 0.29 (note that the BF_10_ values are based on the assumption of a positive correlation for the alternative hypothesis).

## Discussion

This study aimed to investigate how a given LTM representation of object meaning can benefit the short-term retention of simple, geometrical stimuli, while at the same time controlling for possible confounds that could result from the perceptual variability of the presented stimuli. To this end, three sets of “flag” stimuli were presented, which either carried meaning (real flags), or were equally complex, but without conveying meaning (fake flags), or which provided a simple stimulus baseline (unicolor flags). The results showed that the three stimulus types indeed varied in terms of their inherent information load (as estimated from the slopes in the visual search task; see Fig. 2B): the search slopes (averaged across the main and control experiments) were higher for more complex color-shape configurations (real and fake flags: 21 and 24 ms/item, respectively) than for the unicolor stimuli (9 ms/item).^3^ This increase of information load also led to a concurrent reduction of the overall VWM capacity, as evidenced by reduced K estimates (and associated accuracy measures; see Fig. 2A) for fake flags (2.9) as compared to unicolor flags (3.6) – thus, essentially replicating previous findings showing that object complexity determines VWM capacity (e.g., Alvarez & Cavanagh, 2004). Importantly, the real flags – although revealing similar complexity (i.e., information load) estimates as the fake flags – nevertheless resulted in a K estimate of 3.5, which is comparable to the simple unicolor flags (and substantially higher than the fake flags). This indicates that the real flag’s meaningful configuration facilitated VWM performance, despite presenting a relatively complex stimulus configuration. Note that the VWM capacity increase for real flags is unlikely to have resulted from verbalization strategies, as the concurrent digit memory task would have largely prevented the use of verbal memory resources. Finally, the increase in performance for real flags was directly related to the explicit knowledge about the nationality of the presented stimuli: both measures of (real) flag recognition and recall showed that higher K estimates were associated with higher performance in the flag memory test (while no such relationship was observed for the fake flags). This indicates that enhanced VWM performance was facilitated by the knowledge derived from the meaningful flags.

In sum, the current results show that a reduced VWM capacity for more complex objects can be ameliorated when presenting familiar stimuli that are associated with meaning. This is consistent with findings from several previous studies that everyday objects can be memorized with higher accuracy than artificial, meaningless objects (Asp et al., 2021; Brady et al., 2016; Brady & Störmer, 2020a; Sahar et al., 2020; Stojanoski et al., 2019; Veldsman et al., 2017). However, our results significantly extend these previous reports by further demonstrating that the gains for meaningful stimuli can be dissociated from their inherent information load. That is, we show that the improved VWM for real-world objects, as compared to distorted (scrambled, or warped) control stimuli, is not driven by the amount of variability, or complexity of the to-be-memorized objects; rather, the improvement derives, at least in part, from the LTM-based knowledge representation about these objects (such as the association of a country with a given flag). In this respect, our findings are also consistent with other previous studies demonstrating that familiarity and/or expertise for a specific type of stimulus can enhance VWM (Curby et al., 2009; Jackson & Raymond, 2008; Xie & Zhang, 2017). Our current results augment these findings by showing that this benefit generalizes from specialist knowledge of some circumscribed object domains (pokemóns, cars, famous faces) to simple, geometrical color-shape configurations (such as flags).

A potential account to explain our results would assume that encoding of a given stimulus into VWM automatically activates LTM knowledge traces stored about this object. For instance, VWM involves a widely distributed neuronal network that includes sensory, parietal, and prefrontal cortices (for review, see Christophel et al., 2017), where an item representation in VWM might be based on the activation of already-existing long-term memories (Cowan, 1999; Postle, 2015) derived from ventral visual areas that code semantic object information (e.g., Stojanoski et al., 2019). Moreover, the retention of items in VWM has been shown to elicit comparable activation patterns to the LTM recall of stored associations about the same objects (Ranganath et al., 2004). In addition, the recognition of previously encountered stimuli is related to early activations in frontal and parietal cortices (Ranganath & Paller, 1999). VWM and LTM thus appear to be tightly linked, and these associations could help overcome basic capacity limitations to improve behavioral performance.

Somewhat unexpected was the finding that VWM performance was substantially improved for all stimulus types with prolonged encoding durations. While previous work had already shown that VWM may improve with extended encoding times (Eng et al., 2005), Brady et al. (2016) recently reported an encoding-time-dependent improvement of VWM performance for meaningful natural scene stimuli in particular. However, it should be noted that even in that study, VWM capacity estimates improved (at least numerically) for colored squares, too (see also Quirk et al., 2020). It would thus appear that the previously reported lack of a significant increase of VWM performance with display duration may have been owing in part to comparisons of only relatively short encoding durations (e.g., Luck & Vogel, 1997; Nie et al., 2017). And inconsistent findings could have been caused partly by variations in similarity, which may impact the detectability of a given change across different object types (Brady & Störmer, 2021; 2020). However, overall, our findings support the results of Eng et al. (2005) in showing that extended encoding times may lead to a general increase of VWM capacity estimates, irrespective of stimulus type.

In sum, the current results indicate that severe capacity limitations in VWM with simple geometric stimuli (e.g., color-shape configurations) can be substantially reduced by providing meaningful stimuli that trigger LTM associations. Importantly, this knowledge-dependent “nationality” benefit (with the current flag-type stimuli) occurs independently of variations of object complexity, or encoding duration. Together, the results show that VWM can be enhanced by knowing what is remembered.

## Footnotes

1 The configurations of the fake flags were designed not to resemble any known flag. Nevertheless, it remains a possibility that these fake flags still evoked some residual association to a given memory representation, which could in turn have facilitated or interfered with performance to a minor extent.

2 Analogous analyses performed separately on the hit and false-alarm rates revealed the same pattern of results as reported for the (% correct) accuracy data and for the K values: hits were increased and false alarms reduced with longer presentation times and for the real and unicolor (vs. the fake) flag types.

3 The search slope measures may not solely reflect the information load associated with a given object. Rather, visual search may additionally be influenced by memory, such as the retention of a target template that biases search (Chelazzi et al., 1993). This memory-based influence upon search might explain why the slopes were marginally shallower for real flags than for fake flags, reflecting a residual effect of meaning upon search (which was evident to some extent in the main experiment).

## Declarations

### Data availability

The data, experimental code and stimulus materials presented in the current study are available from the corresponding author upon reasonable request.
